# Effects of Progressive Aerobic Training on Executive-Reward Network Connectivity and Symptoms of Internet Gaming Disorder: Randomized Controlled Trial

**DOI:** 10.2196/83597

**Published:** 2025-11-28

**Authors:** Shaoyu Cui, Xuefeng Xu, Xin Luo, Meiting Wei, Guang-Heng Dong

**Affiliations:** 1 Department of Psychology, Yunnan Normal University, 1st Lianda Street, Chenggong District, Kunming, Yunnan Province, 650500, China, 86 15867949909; 2 Faculty of Education, Yunnan Normal University, 1st Lianda Street, Chenggong DistrictKunming, Yunnan Province, China

**Keywords:** functional connectivity, internet gaming disorder, progressive aerobic training, randomized controlled trial, neural mechanism, brain dysfunction

## Abstract

**Background:**

Internet gaming disorder (IGD) causes neurocognitive deficits and brain dysfunction. Progressive aerobic training (PAT) seems more practical. However, its effect on IGD and the underlying neural mechanism remains unclear.

**Objective:**

This preregistered, randomized controlled, single-blind study examined the efficacy of a novel non-pharmacological intervention by elucidating the neurocognitive mechanisms in IGD.

**Methods:**

A total of 72 participants with IGD (meeting the DSM-5 criteria for IGD, Internet Addiction Test [IAT] score >50, and no comorbid conditions) were recruited and randomly assigned to a PAT group (received 12 weeks of PAT with the intensity dynamically adjusted based on real-time performance) or a free training (FT) group (completed sessions of equal duration but without a progressive structure, allowing free choice of exercise mode and intensity). No intervention-related adverse events were reported. Sixty-four participants completed the experiment (PAT: 33; FT: 31), including pretreatment and posttreatment functional magnetic resonance imaging scans and 20 PAT sessions in a month. Regional homogeneity and degree centrality are calculated; the overlapping brain regions were used as seed points for functional connectivity (FC) analysis. The correlation between FC and behavioral data and neurotransmitters was also evaluated.

**Results:**

The PAT group demonstrated a significantly greater reduction in IAT scores compared to the FT group (*t*_32_=4.333, *P_bonf_*<0.001, Cohen *d*=0.754, 95% CI 0.362-1.137), accompanied by a specific reduction in game craving within the PAT group (*t*_32_=2.278, *P_bonf_*=0.03, Cohen *d*=0.397, 95% CI 0.045-0.851]). FC analysis revealed that PAT significantly enhanced FC within the executive control network (ECN), increasing connectivity between the right medial superior frontal gyrus (R-mSFG) and key regions, including the left postcentral gyrus (*F*_1, 62_=7.95, *P*=.006), bilateral superior parietal gyrus (right: *F*_1, 62_=5.68, *P*=.02; left: *F*_1, 62_=8.85, *P*=.004), and left inferior frontal gyrus (*F*_1, 62_=11.37, *P*=.001). PAT also strengthened ECN-reward network (RN) integration, enhancing FC between the R-mSFG and bilateral insula (right: *F*_1, 62_=11.41, *P*=.001; left: *F*_1, 62_=7.94, *P*=.006) and left substantia nigra (*F*_1, 62_=10.60, *P*=.002). These neural changes were behaviorally relevant, as pretest game craving positively correlated with post-intervention FC strength between the R-mSFG and left postcentral gyrus (*r*=0.36, *P*=.04, 95% CI 0.02‐0.63) and right precentral gyrus (*r*=0.40, *P*=.02, 95% CI 0.07‐0.65). Furthermore, the FC changes were significantly associated with cannabinoid (CB1) (*P*=.003) and metabotropic glutamate (mGluR5) receptor distributions (*P*=.005).

**Conclusions:**

This study demonstrates the efficacy of a progressive and adaptive PAT intervention in reducing IGD severity, moving beyond static protocols by dynamically tailoring intensity to individual performance. The therapeutic effect may be mediated by modulating the functional connectivity between the ECN and the RN, potentially enhancing top-down control. These results, supported by the correlation between FC, behavior, and neurotransmitter systems, indicate that PAT represents a promising non-pharmacological intervention approach worthy of further investigation.

## Introduction

 Internet gaming disorder (IGD) has emerged as a severe mental health issue that needs to be addressed [1,2]. Both the *DSM-5* (*The Diagnostic and Statistical Manual of Mental Disorders, Fifth Edition*) and ICD-11 (*The International Classification of Diseases, 11th Revision*) define this disorder as a repetitive or continuous gaming behavior pattern that lasts for at least 12 months, unless severe symptoms occur [3-5]. IGD can lead to severe negative consequences for players, including neurocognitive deficits, impaired executive functioning, and emotional dysregulation [6,7], which in turn generate physical, psychological, and behavioral effects on players.

 Neuroimaging studies have identified significant alterations in functional brain networks associated with IGD [8], including impaired executive control over gaming cravings and strong gaming cravings in reward processing-related brain regions [9,10]. Multiple studies have observed relatively decreased functional connectivity (FC) between executive-control–related and reward-related regions [11,12]. One study found that the IGD group showed decreased FC between the right superior frontal gyrus (SFG) and right posterior insula compared to controls [13]. Another found that the IGD group showed significantly decreased FC values between the right middle frontal gyrus (as well as dorsolateral prefrontal cortex; DLPFC) and the right anterior cingulate cortex [11,14]. Moreover, reduced FC strength revealed weakened connectivity in executive-control–related regions, including SFG-DLPFC [15] and DLPFC-postcentral gyrus [16].

 Traditional interventions for IGD have focused mainly on psychological, pharmacological, and exercise-based therapies [17]. These treatment strategies need to perform by professional trained psychiatrist and usually expensive and hard to perform. Comparing with these treatment strategies, the exercise-based interventions may be more practical [18] as it is widely acceptable and easily performed in daily life. Exercise intervention, such as the aerobic exercise, is known to increase brain-derived neurotrophic factor, and the increased brain-derived neurotrophic factor enhances cognitive function by modifying neurotransmission, stimulating neurogenesis, and promoting synaptic plasticity in the brain [19]. Aerobic exercise has been proven to restore and regulate nerve cells, enhancing their adaptability to external variations, and optimizing cognitive abilities, executive functions, and inhibitory control, while fostering positive effects and mitigating negative impacts [20-22]. In previous studies on aerobic training for IGD, Liu et al [23] found through systematic review and meta-analysis that mind–body exercises like Tai Chi and Qigong can significantly improve executive function. In a study of training on brain function in methamphetamine addicts, aerobic training temporarily altered the activation of neural networks in the reward system of drug-dependent individuals, effectively inhibiting their drug craving [24]. Rensburg et al [25] explored the effects of training on the brain during withdrawal and suggested that individuals exercising 15 hours after quitting smoking show significant reductions in smoking craving and significant activation of the brain reward system. Meanwhile, physical training normalizes dopaminergic and glutamatergic transmissions [26]. Cannabinoids are thought to play a crucial role in mediating the mood-regulating effects of exercise [27], while mGluR5 is pivotal for the central reward processing in addicted individuals [28]. These evidence supports the hypothesis that aerobic training mitigates IGD by restoring the functional balance between the reward and control networks, a process potentially mediated by its synergistic effects on dopaminergic, glutamatergic, and endocannabinoid systems.

 Although extensive research underscores the therapeutic potential of exercise, the neurofunctional effects of fixed, progressive aerobic training (PAT) regimens in individuals with IGD remain uncharted in the published literature. A study on morphine-addicted rats shows that compared to the fixed aerobic protocol, progressive aerobic exercise significantly outperformed in reversing morphine-induced impairments in memory, memory consolidation, and locomotor activity [29]. One study suggests that 24-week PAT could significantly improve the anxiety symptoms, psychological pressure and sleep state of male drug addicts [30]. However, the specific impact of PAT on the neural mechanism with IGD remains unclear.

 Both regional homogeneity (ReHo) and degree centrality (DC) are two main resting-state functional magnetic resonance imaging metrics in a way of voxel-wise whole-brain analysis [31,32]. ReHo is a voxel-based measure of brain activity that estimates the degree of synchronization between the time series of a given voxel and its nearest neighbors [33,34]. The validity of this measure is based on the premise that intrinsic brain activity primarily reflects the coordinated activation of voxel clusters rather than isolated single voxels. Consequently, higher ReHo values are thought to represent greater synchronization of local field potentials associated with neuronal activity in the human brain [35]; DC considers Pearson correlation coefficient as metric for FC estimation between each pair of voxels, assigning to each voxel the global number of functional connections between it and all other voxels across the brain [36]. In its more diffuse implementations, it involves the use of a threshold over which a pair of voxels is considered connected [37,38]. The combination of ReHo and DC captures both local and remote neural synchronization [39], demonstrating superior performance over traditional methods for selecting regions of interest (ROIs) in resting-state functional magnetic resonance imaging (fMRI) [40,41].

 While IGD research has not employed PAT intervention, dance as a sport enhances frontal-striatal circuit connectivity in individuals with IGD [23]. Research on heroin addiction further suggests that physical exercise serves as an effective non-pharmacological intervention, capable of improving patients’ executive control function [42]. Therefore, this study evaluated the efficacy and neural mechanisms of PAT on IGD through FC and correlation analysis. We hypothesized that PAT would reduce the severity of game craving and addiction severity in IGD by altering the FC of brain regions involved in executive control network (ECN) and reward network (RN).

## Methods

### Trial Design and Involvement

No patient or public involvement was incorporated into the design, conduct, reporting, or dissemination of this research. This was a prospective, randomized, controlled, single-blind trial with two parallel groups. The research was conducted at the Department of Psychology of Yunnan Normal University in Kunming, China, from November 1, 2024, to December 1, 2024. The study design and reporting adhered to the CONSORT 2025 guidelines, specifically following the CONSORT checklist for reporting information in randomized trials (Checklist 1) [43]. The trial proceeded to its planned completion without early termination.

### Ethical Considerations

The study protocol was reviewed and approved by the Human Investigation Committee (Institutional Review Board) at Yunnan Normal University (Approval No: yunnuethic2024-044; registered prospectively on 2024-10-14). No changes were made to the trial protocol after its commencement. Written informed consent was obtained from all participants prior to their enrollment in the study. To ensure participant privacy and confidentiality, all collected data were immediately deidentified following collection. Participants were assigned anonymous codes, and all analyses were performed using these coded identifiers. No personally identifiable information is included in this manuscript or any supplementary materials. All participants who completed the entire study protocol, including pretest and posttest assessments and all training sessions, received financial compensation of 400 RMB (approximately US $56.29).

### Participants

**Figure 1.**
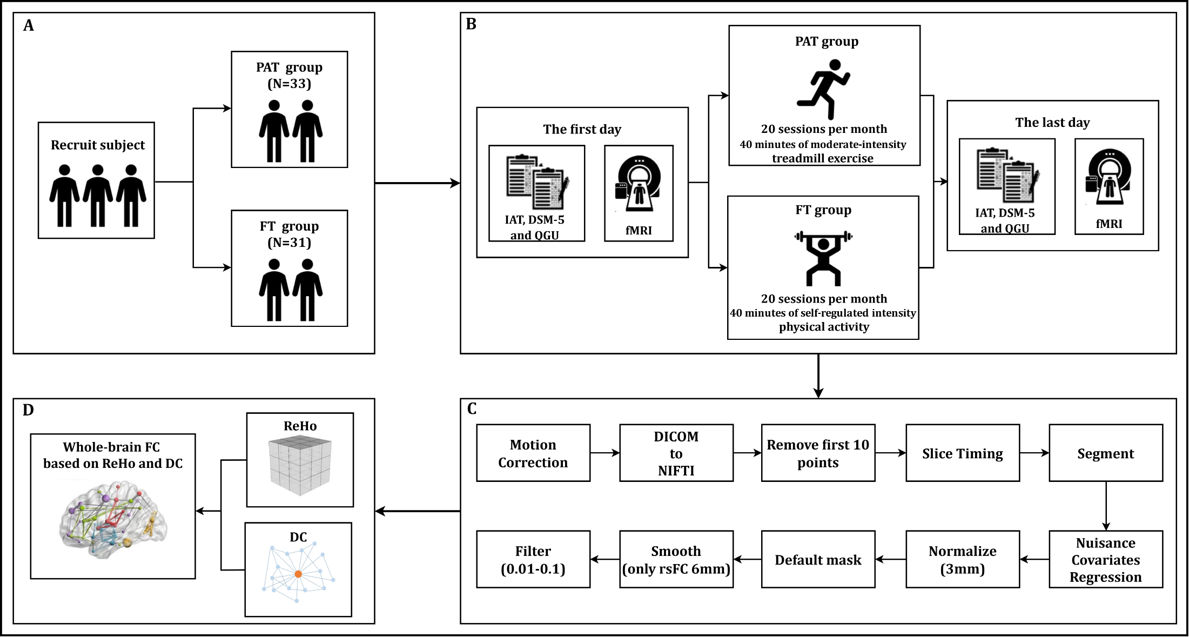
Experimental timeline and procedures for the randomized controlled trial of progressive aerobic training versus free training in individuals with internet gaming disorder. (**A**) Subject; (**B**) treatment processing; (**C**) data preprocessing; (**D**) data analysis. PAT: progressive aerobic training; FT: free training; IAT: internet addiction test; DSM: *Diagnostic and Statistical Manual of Mental Disorders*; QGU: questionnaire for gaming urges; fMRI: functional magnetic resonance imaging; ReHo: regional homogeneity; DC: degree centrality; FC: functional connectivity.

Figure 1 shows the process and sequence of the study. This study initially recruited 72 participants with IGD. Eight participants dropped out during the intervention training, resulting in a final cohort of 64 participants (PAT: n=33; FT: n=31). No missing data were encountered (no additional prespecified or post-hoc analyses were performed).

We recruit patients with IGD from schools through advertisements to obtain treatment opportunities for improving mental health. Researchers require interested participants to participate in the internet addiction test (IAT) online [44], which has been proven to effectively diagnose internet addiction. Participants with scores greater than 50 are required to undergo an offline professional interview with a psychiatrist. Select eligible research subjects using *DSM-5* criteria [45], and Mini International Neuropsychiatric Interview (MINI) [46]. 

The criteria for inclusion in participants are as follows: (1) IAT score >50; (2) *DSM-5* score >5; (3) active engagement in online gaming exceeding 1 year; (4) BMI within normal to overweight range (15≤BMI≤28); (5) no cognitive impairment and no other psychiatric disorders confirmed by MINI; (6) no previous treatment for IGD; (7) no experience of regular use of any psychotropic drugs. The exclusion criteria for participants were as follows: (1) majors related to sports; (2) *DSM-5* score <5; (3) the body contains metals. All participants who met the inclusion criteria were randomly assigned to either the PAT group or the FT group. Detailed demographic data for the two groups are shown in Table 1.

**Table 1.** Demographic and clinical characteristics of participants with internet gaming disorder at baseline.

Characteristic	PAT^a^ group, n=33	FT^b^ group, n=31	*t* test^c^ *(df)*	*P* value
Sex, n (%)			—^d^	—^d^
Male	11 (33.33)	13 (41.93)		
Female	22 (66.66)	18 (58.07)		
Age, mean (SD)	19.97 (1.36)	19.87 (1.61)	0.266 (62)	.79
IAT^e^ score, mean (SD)	62.88 (12.196)	62.94 (13.878)	−0.017 (62)	.99
DSM-5^f^ score, mean (SD)	5.24 (2.092)	5.19 (2.088)	0.093 (62)	.93
QGU^g^ score, mean (SD)	34.48 (16.333)	30.13 (17.957)	1.016 (62)	.31

a PAT: progressive aerobic training.

b FT: free training.

c The *t* tests reported in the table are all two-tailed independent samples tests.

d not applicable.

e IAT: internet addiction test.

f DSM-5: Diagnostic and Statistical Manual of Mental Disorders-5.

g QGU: Questionnaire for Gaming Urges.

### Intervention Processes

In this study, the American College of Sports Medicine guidelines were used to develop an aerobic training program with an aerobic intensity of 46% in the first phase and 55% in the second phase, as well as a training duration of 40 minutes per session for a total of 20 sessions in 1 month [47-49]. The PAT group trained only on the treadmill at the speed calculated from the step test [50]. The specific training process and related contents are shown in Multimedia Appendix 1. The interventions were delivered by research assistants who were trained in the specific protocols for both the PAT and FT groups to ensure consistency and safety.

### Adverse Events

As adverse events were not systematically assessed, none were reported during the trial.

### Sample Size

A comparison between study completers and dropouts showed no significant differences in key demographic or clinical measures at baseline (Multimedia Appendix 1), indicating that attrition bias is unlikely to have substantially influenced the primary findings. This trial involved no interim analyses or stopping guidelines.

Using G-power, setting the effect size at *f*=0.25 and α error probability at 0.05, we estimated the required sample size at 66. The current sample is a bit lower than the required 66.

### Randomization to the PAT or FT Group

Participants were randomly allocated to either the PAT group or the FT group using simple randomisation with a 1:1 allocation ratio. The random allocation sequence was generated by an independent researcher not involved in participant recruitment or intervention. The sequence was concealed using sequentially numbered, opaque, sealed envelopes. The principal investigator enrolled participants, and a research assistant then assigned them to groups by opening the next envelope in the sequence.

### Blinding

The participants were indeed blinded to the existence of the other experimental group. Both groups were in the same training room but did not know the existence of the other group (the PAT group trained from 14:00-19:00; the FT group trained from 20:00-22:00). Research assistants conducting assessments were aware of group allocations due to the practical constraints of implementing the intervention protocols.

### Behavioral Measurements

The current analysis is a per-protocol analysis; only subjects who finished the pretest, 20-time training, and posttest were included. Before the first training and after the entire training period, all participants’ addiction levels were measured based on the IAT, *DSM-5* criteria, and craving scores. Craving was measured with the Questionnaire for Gaming Urges, adapted from the Tiffany Questionnaire for Smoking Urges [51]. Meanwhile, before the first training session, after 10 interventions and all 20 interventions, we conducted a 3-minute step test [50] on all participants to obtain the maximum oxygen uptake for dynamically adjusting the intensity of PAT.

### Magnetic Resonance Imaging Data Acquisition

The magnetic resonance imaging data were acquired using a Siemens Trio 3T scanner. The specific parameters used were as follows: repetition time=2000 ms, 54 interleaved slices, echo time=30 ms, thickness=3.0 mm, flip angle=90°, field of view=220 mm × 220 mm, and matrix=64×64. Head motions were minimized by filling the empty space around the subjects’ heads with sponge and fixing their lower jaws with tape.

### Data Preprocessing

The preprocessing of the rs-fMRI data was completed according to Statistical Parametric Mapping principles [52] using SPM12 [53], and data processing and analysis were performed using Data Processing & Analysis of Brain Imaging (DPABI) [54,55]. The preprocessing of the functional rs-fMRI data included the following steps: (1) the quality of the raw rs-fMRI data for all the participants was examined; (2) the raw data were transformed from the Digital Imaging and Communication in Medicine (DICOM) format to the Neuroimaging Informatics Technology Initiative (NIFTI) format; (3) the initial 10 volumes were discarded to maintain magnetic field stabilization; (4) slice timing was performed; (5) realignment was performed (excluding subjects with maximum head motion >2.5 mm or rotation >2.5°); (6) regressing nuisance covariates were included, including the signal noise of white matter, cerebrospinal fluid, and Friston-24 head motion parameters [56]; (7) the data were normalized using the standard procedure within the DPABI pipeline, which registers the images to the MNI-152 template; (8) the data were filtered by a bandpass filter with a frequency range of 0.01 to 0.1 Hz before the calculation of ReHo and DC; and (9) the data were smoothed using a Gaussian kernel with a full width at half maximum of 6 mm.

### ROI Selection

We first computed ReHo and DC values for both the PAT and FT groups using pretest and posttest measurements. Repeated-measure ANOVAs were then conducted for two groups using the DPABI Viewer toolbox, with a statistical threshold set at *P*<.01 and a cluster size threshold of >100 voxels (corrected for the false discovery rate).

### Statistical Analysis

The prespecified primary outcome was the change in the Questionnaire for Gaming Urges score from baseline to post-intervention. Secondary outcomes included other behavioral measures and functional connectivity measures (including their correlation with normative neurotransmitter maps).

We conducted whole-brain FC analysis using the ROI obtained by overlapping DC and ReHo. Then, significant brain region data were extracted with the DPABI toolbox for repeated measures ANOVA analysis in JASP (Version 0.19.3) [57,58], applying a Bonferroni correction (*α*=.05, corrected for multiple comparisons). The correlation analysis of behavioral data was also completed using JASP. Subsequently, we performed a correlation analysis between FC results and neurotransmitter levels using JuSpace 1.5 (10,000 permutations, *P*<.05, with correction for the false discovery rate) [59]. The analysis demonstrates a spatial correspondence between our FC findings and the normative neurotransmitter maps, rather than a correlation with measured individual neurochemical levels.

## Results

### Overview

**Figure 2.**
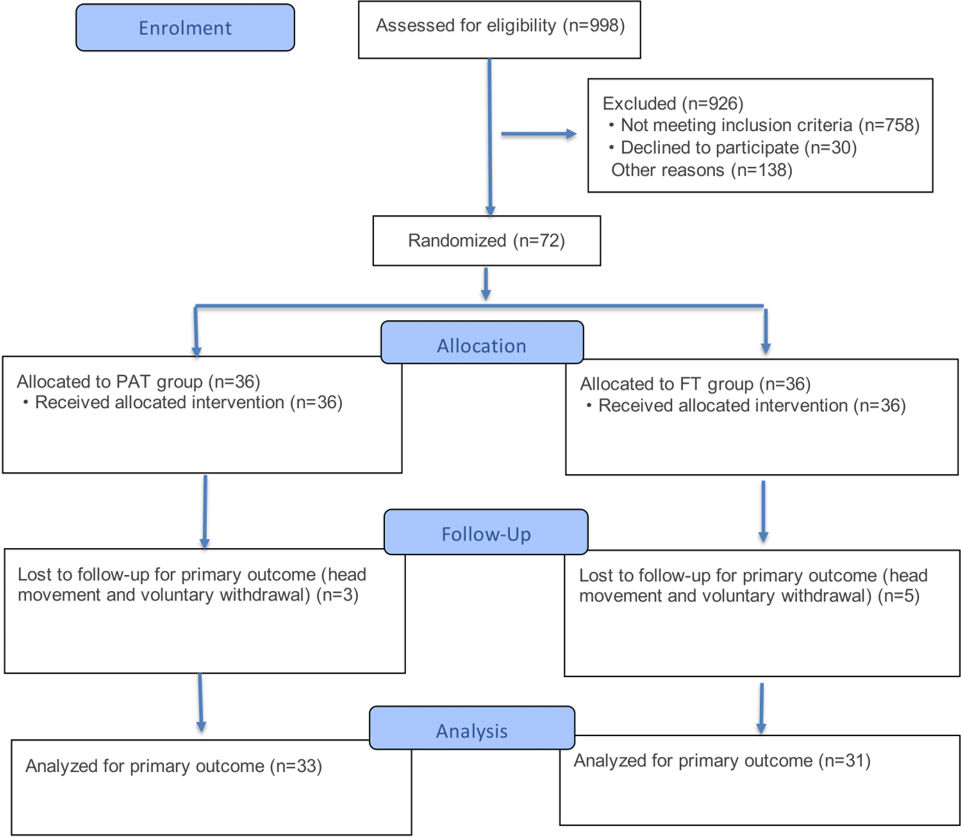
CONSORT flow diagram of participant screening, randomization, and analysis in a trial of progressive aerobic training (PAT) versus free training (FT) for internet gaming disorder.

The following analysis was conducted with 64 participants who completed the interventions without receiving other therapies, as shown in Figure 2. Consistent with the methods, no intervention-related adverse events were observed, and no additional prespecified or post-hoc analyses were performed.

### Behavioral Measures

**Figure 3.**
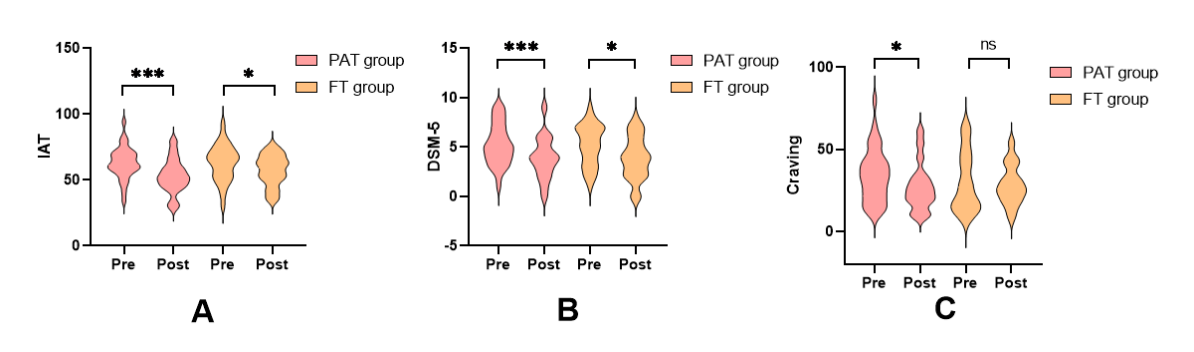
Behavioral changes in internet gaming disorder following progressive aerobic training (PAT) versus free training (FT). DSM, Diagnostic and Statistical Manual of Mental Disorders, version 5; Pre, pretest before training; Post, posttest after training. * *P*<.05, ** *P*<.01, *** *P*<.001, ns: no significance.

We performed repeated measures ANOVA on game craving in the PAT and FT groups and found that the interaction was not significant. Then, paired-samples *t* tests were conducted to compare pretest and posttest gaming cravings in the PAT group and the FT group. The results revealed a significant reduction in gaming cravings in the PAT group (*t*_32_=2.278, *P_bonf_*=0.03, Cohen *d*=0.397, 95% CI 0.045-0.851), whereas no significant change was observed in the FT group (*t*_30_=0.481, *P_bonf_*=1, Cohen *d*=0.086, 95% CI −0.306 to 0.502). Compared to the FT group (*t*_30_=2.277, *P_bonf_*=0.03, Cohen *d*=0.409, 95% CI 0.039-0.773), the PAT group showed a significantly greater reduction in the IAT score (*t*_32_=4.333, *P_bonf_*<0.001, Cohen *d*=0.754, 95% CI 0.362-1.137). This finding was consistent with *DSM-5* scores (Figure 3).

### Functional Connectivity Results

#### FC Results Within the ECN

Repeated-measures ANOVA showed a significant interaction between the changes in FC between the right medial superior frontal gyrus (R-mSFG) and left postcentral gyrus (*F*_1, 62_=7.953, *P*=.006), bilateral superior parietal gyrus (SPG; right: *F*_1, 62_=5.675, *P*=.02; left: *F*_1, 62_=8.853, *P*=.004), left inferior frontal gyrus (*F*_1, 62_=11.373, *P*=.001), right precentral gyrus (*F*_1, 62_=7.325, *P*=.009), right middle cingulate gyrus (*F*_1, 62_=6.659, *P*=.012) in both groups before and after intervention.

Subsequent post-hoc analysis demonstrated that the PAT group exhibited significantly enhanced FC between the R-mSFG and left postcentral gyrus (*t*_62_=−2.726, *P_bonf_*=0.008, Cohen *d*=−0.633, 95% CI −1.268 to 0.002), bilateral SPG (right: *t*_62_=−2.417, *P_bonf_*=0.019, Cohen *d*=−0.525, 95% CI −1.126 to 0.076; left: *t*_62_=−2.861, *P_bonf_*=0.006, Cohen *d*=−0.693, 95% CI −1.358 to −0.027), left inferior frontal gyrus (*t*_62_=−3.254, *P_bonf_*=0.002, Cohen *d*=−0.642, 95% CI −1.202 to −0.082), right precentral gyrus (*t*_62_=−2.26, *P_bonf_*=0.027, Cohen *d*=−0.517, 95% CI −1.141 to 0.107), and right middle cingulate gyrus (*t*_62_=−2.42, *P_bonf_*=0.018, Cohen *d*=−0.567, 95% CI −1.221 to 0.087). No such effects were observed in the FT group.

#### FC Results Between the ECN and the RN

Repeated-measures ANOVA showed a significant interaction between the changes in FC between the R-mSFG and bilateral insula (right: *F*_1, 62_=11.408, *P*=.001; left: *F*_1, 62_=7.94, *P*=0.006) and left substantia nigra (*F*_1, 62_=10.604, *P*=.002) in both groups before and after intervention.

Subsequent post-hoc analysis confirmed that the PAT group showed significantly enhanced FC between the R-mSFG and bilateral insula (right: *t*_62_=−3.372, *P_bonf_*=0.001, Cohen *d*=−0.59, 95% CI −1.089 to −0.092; left: *t_62_*=−2.761, *P_bonf_*=0.008, Cohen *d*=−0.611, 95% CI −1.233 to 0.01) and left substantia nigra (*t_62_*=−3.157, *P_bonf_*=0.002, Cohen *d*=−0.778, 95% CI −1.476 to −0.080). However, no significant changes were detected in the FT group.

#### Correlation Analyses Between FC Results and Behavioral Data

**Figure 4.**
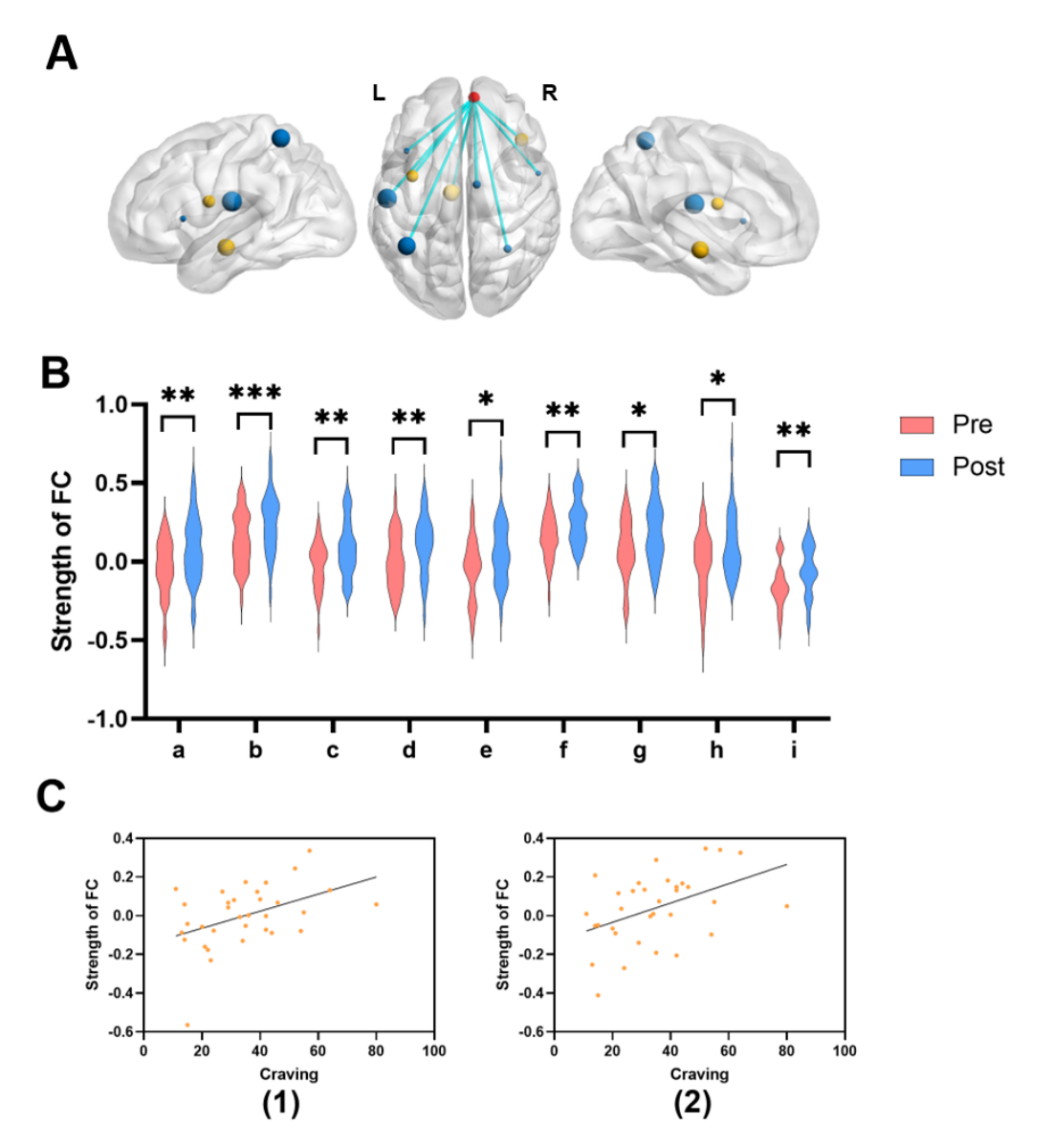
Functional connectivity (FC) alterations and their association with game craving following progressive aerobic training. (**A**) Visualization of the region of interest and functional connectivity. The size of the ball corresponds to the t value obtained from the simple effect. The red ball indicates the right medial superior frontal gyrus (R-mSFG); the blue ball indicates the executive control network including the left postcentral, bilateral superior frontal gyrus (SFG), left inferior frontal gyrus (IFG), right precentral gyrus, and right middle cingulate gyrus (MCG); and the yellow ball indicates the reward network including the bilateral insula and left substantia nigra. (**B**) Whole brain–based functional connectivity (FC) results. The x-axis shows the different functional connections: a, the R-mSFG to the left postcentral gyrus; b, the R-mSFG to the right insula; c, the R-mSFG to the left superior parietal gyrus (SPG); d, the R-mSFG to the left IFG; e, the R-mSFG to the right precentral gyrus; f, the R-mSFG to the left substantia nigra; g, the R-mSFG to the right MCG; h, the R-mSFG to the right SPG; i, the R-mSFG to the left insula. Pre, pretest before the training; Post, posttest after the training. * *P*<.05, ** *P*<.01, *** *P*<.001. (**C**) Correlation analysis between FC results and levels of game craving. (1) Correlation results between game craving and FC strength between the R-mSFG and left postcentral gyrus in the progressive aerobic training group. (2) Correlation results between game craving and FC strength between the R-mSFG and the right precentral gyrus.

The ReHo analysis revealed significant activation in the R-mSFG, while the DC analysis identified significant regions in the bilateral medial superior frontal gyrus and the left precuneus. Subsequently, we performed an overlap analysis between the ReHo and DC results to identify common brain regions. At *P*<.01, the R-mSFG emerged as the only significant overlapping region (Figure 4). Based on these findings, we selected the R-mSFG as the ROI for subsequent whole-brain FC analysis. In the PAT group, a significant positive correlation (*r*=0.359, *P*=.04, 95% CI 0.018-0.625) between pretest game cravings and FC strength between the R-mSFG and left postcentral gyrus was found. The same result was found for the R-mSFG to the right precentral gyrus (*r*=0.399, *P*=.021, 95% CI 0.065-0.653; Figure 4).

#### Correlation Analyses Between FC Results and Neurotransmitters

In the pretest of the PAT group, compared to the posttest results, significant correlations were observed between the spatial distribution of CB1 receptors (*P*_*bonf*_=.003), mGluR5_rosaneto receptors (*P*_*bonf*_=.005), and mGluR5_dubois receptors (*P*_*bonf*_=.005) based on FC changes in the R-mSFG (Multimedia Appendix 2).

## Discussion

### Principal Findings

In this study, we evaluated the efficacy and neural mechanisms of the PAT intervention for IGD. The results indicate that PAT was associated with reduced severity of addiction and craving levels, alongside alterations in the FC within the ECN and between the ECN and the RN.

### PAT Reduces Addiction Severity and Gaming Cravings in IGD

Following the PAT intervention, participants in the PAT group exhibited markedly lower gaming cravings and decreased addiction severity relative to the AT group. Although PAT has not been used in previous literature for the treatment of IGD, previous studies on alcohol and nicotine dependence have shown that PAT training can reduce cravings for addictive substances and the severity of addiction [60,61]. Therefore, this study found decreased game craving and addiction severity after PAT, indicating that PAT is an effective intervention for patients with IGD, significantly mitigating the symptoms of IGD.

### PAT Strengthened FC Within the ECN

This study found enhanced FC between the prefrontal cortex (mSFG and IFG), SPG, middle cingulate gyrus, and postcentral and precentral gyrus in IGD after PAT. The prefrontal cortex and postparietal cortex are implicated in a wide range of cognitive functions, particularly cognitive control [62,63]. The SPG has a crucial role in visual perception, spatial cognition, and attention [64,65]. Resting-state fMRI has found that compared to healthy controls, patients with IGD showed decreased FC in the ECN (including several parts of the frontoparietal cortex) [66,67], indicating impaired executive functioning. The findings of this study demonstrate that PAT contributed to enhancing the functional integrity of ECN implicated in IGD. The precentral gyrus and postcentral gyrus are the key regions of sensorimotor networks associated with integrating sensorimotor information and coordinating physical movement [68,69]. Although the precentral gyrus is not part of the ECN, executive control processes frequently require integrating sensory information transmitted from the precentral gyrus to achieve efficient behavioral regulation [70]. Therefore, this study found that the FC within the ECN was significantly enhanced following PAT, indicating that PAT may help normalize sensorimotor dysfunction of IGD, thereby contributing to the restoration of executive control and emotional regulation capabilities.

### PAT Strengthened FC Between the ECN and the RN

The present study found enhanced FC between the mSFG, bilateral insula, and substantia nigra. The insula plays a pivotal role in the RN, engaging in reward processing and the regulation of motivated behaviors by integrating sensory and emotional information. It influences decision-making and is closely associated with mental health [71,72]. In the context of addiction, insula dysfunction may contribute to heightened cravings and impulsive behaviors, disrupting the balance between impulse control and reward processing [73]. The substantia nigra, a core component of the basal ganglia, plays a pivotal role in reward-related processing [74]. Meanwhile, the superior frontal gyrus, a key region within the ECN and a default mode network seed region, is critical for top-down cognitive control, including decision-making, selective attention, inhibitory control, and emotion regulation [75,76]. Consistent with prior research, individuals with IGD exhibit reduced FC between the ECN and RN compared to healthy controls [77-79]. Therefore, this study revealed that the FC between the ECN and RN was significantly enhanced following PAT, indicating that PAT may weaken gaming cue reactivity and strengthen top-down regulation of reward processing in patients with IGD.

### Association Between PAT and Molecular Systems in IGD

Few studies have correlated the neuroimaging changes of IGD with the activity of microscopic neurotransmitters [80,81]. This study found an association between the FC and activity maps of CB1 and mGluR5 in the PAT group. CB1 cannabinoid receptors are located presynaptically in multiple brain regions, including reward-related structures like the caudate-putamen, substantia nigra, and globus pallidus [82,83]. It is well-established that aerobic exercise is a potent trigger for the release of endogenous cannabinoids, such as anandamide, in the brain [84]. The primary central receptor for these endogenous cannabinoids is the CB1 receptor. Therefore, our finding that functional connectivity changes correlate with the spatial distribution of CB1 receptors provides a direct neurobiological substrate for the effects of PAT. We posit that the PAT in our study enhanced the signaling of endogenous cannabinoids, which in turn modulated neural circuitry in CB1-rich brain regions that are critically involved in IGD pathology.

Furthermore, the significant correlation with the mGluR5 map is highly informative. The mGluR5 is highly expressed in key addiction-related regions, including the nucleus accumbens, cerebral cortex, hippocampal formation, and amygdala [28]. Both CB1 and mGluR5 are considered to exhibit functional interactions and are known to form heteromeric complexes that regulate synaptic plasticity [85,86]. This provides a compelling rationale for our concurrent findings regarding both receptors. We thus hypothesize that the therapeutic action of PAT is mediated not by a single system, but by the coordinated modulation of CB1 and mGluR5 signaling, which promotes adaptive synaptic plasticity to normalize the dysregulated neural circuitry underlying IGD.

### Limitations

This study has several limitations. First, while our FC analysis revealed altered connectivity between key brain regions, it did not elucidate the directionality of these changes. Future studies could employ effective connectivity analysis to determine the causal interactions underlying these FC modifications. Second, the FT group exhibited a slight therapeutic effect on IGD symptoms, suggesting a potential placebo effect. To address this limitation, future research could incorporate multiple control groups to better isolate the specific effects of PAT. Third, our sample was recruited solely from schools, which may limit the generalizability of the findings to older individuals with IGD. Fourth, it should be noted that potential confounding factors, such as general fitness improvement, social interaction during training, and participant expectancy effects, may have contributed to the observed outcomes. Finally, the modest sample size in this study may limit the generalizability of the findings. Future research with a larger, multicenter cohort, incorporating long-term follow-ups, would enhance the statistical power and robustness of the findings.

### Conclusions

This study provides evidence for the beneficial effects of PAT on IGD symptoms. A key innovation of this trial is its implementation of a progressive aerobic intervention, which dynamically adjusted intensity based on the real-time performance of individuals with IGD. This approach moves beyond conventional fixed-intensity protocols to offer a more personalized and potentially more effective therapeutic approach. At the cognitive-neural level, our findings suggest that PAT may enhance executive control function in patients with IGD concurrently with alterations in its regulatory influence over the reward system. These neural improvements were substantiated by significant correlations between neurofunctional markers and behavioral measures. The results offer insights into the neural mechanisms associated with PAT’s positive outcomes in IGD and provide a foundation for future clinical intervention research.

## Supplementary material

10.2196/83597 Multimedia Appendix 1 The specific training process and related contents follow progressive aerobic training versus free training.

10.2196/83597 Multimedia Appendix 2 Correlations between functional connectivity and neurotransmitter systems in the progressive aerobic training (PAT) group for internet gaming disorder. 5-HT, 5-hydroxytryptamine; CB1, cannabinoid 1 receptor; mGluR5, metabotropic glutamate receptor 5. ** *P*<.01.

10.2196/83597 Checklist 1 CONSORT-EHEALTH checklist (V1.6.1).

## Abbreviations

DC: degree centrality
DICOM: Digital Imaging and Communication in Medicine
DLFPC: dorsolateral prefrontal cortex
DPABI: Data Processing & Analysis of Brain Imaging
DSM-5: The Diagnostic and Statistical Manual of Mental Disorders, Fifth Edition
ECN: executive control network
FC: functional connectivity
fMRI: functional magnetic resonance imaging
FT: free training
ICD-11: The International Classification of Diseases, 11th Revision
MINI: Mini International Neuropsychiatric Interview
PAT: progressive aerobic training
R-mSFG: right medial superior frontal gyrus
ReHo: regional homogeneity
RN: reward network
ROI: region of interest
SFG: superior frontal gyrus
SPG: superior parietal gyrus
