# Supplementary material for: Effects of Progressive Aerobic Training on Executive-Reward Network Connectivity and Symptoms of Internet Gaming Disorder: Randomized Controlled Trial
Source: JMIR Serious Games. 2025 Nov 28;13:e83597. doi: 10.2196/83597 (PMC12669919; doi:10.2196/83597)
Supplement: Multimedia Appendix 1 [file games-v13-e83597-s001.docx]

SUPPLEMENTARY MATERIALS

Effects of Progressive Aerobic Training on Executive-Reward Network Connectivity and Symptoms of Internet Gaming Disorder: A Randomised Controlled Trial

| **S1.** The detailed intervention program for different groups | | |
| --- | --- | --- |
| Item | PAT | FT |
| Pre-test | IAT, DSM5, QGU, IPAQ Chinese short questionnaire, 3-minute Mcardle pacing test (to obtain maximal oxygen uptake VO_2max_) and fMRI scan.  Aerobic (at 0% horizontal incline) treadmill speed was obtained from VO_2max_. The formulae are shown in S2. | IAT, DSM5, QGU, IPAQ Chinese short questionnaire, 3-minute Mcardle pacing test (to obtain maximal oxygen uptake VO_2max_) and fMRI scan. |
| First 10 sessions Aerobic intervention | The corresponding treadmill speed was calculated for each subject at 46% of the desired intensity.  Before each aerobic exercise, 10 min of warm-up, 40 min of aerobic exercise on the treadmill, followed by 10 min of muscle stretching activities were performed. | No treadmill speed was specified, allowing subjects to train freely. Warm up for 10 min before each exercise. no restriction on the type of exercise (including running, slow walking, jumping rope, stationary bicycle, resistance equipment, etc.). No restriction on the intensity of training, the subjects decided on their own. The duration of the exercise was not limited, and the duration of a single session was not more than 40 min, followed by 10 min of muscle stretching. |
| Second test maximal oxygen uptake | 3min Mcardle step test (to obtain maximal oxygen uptake VO_2max_) | 3min Mcardle step test (to obtain maximal oxygen uptake VO_2max_) |
| Post 10 aerobic interventions | The corresponding treadmill speed was calculated for each subject according to the maximal oxygen uptake VO_2max_ of the second test, recalibrated at 55% of the desired intensity.  Before each aerobic exercise, 10 min of warm-up and 40 min of aerobic exercise on the treadmill were performed, followed by 10 min of muscle stretching activities. | Equivalent to the first 10 training sessions. |
| Post-test | IAT, DSM5, QGU, IPAQ Chinese short questionnaire, 3-minute Mcardle pacing test (to obtain maximal oxygen uptake VO_2max_) and fMRI scan. | IAT, DSM5, QGU, IPAQ Chinese short questionnaire, 3-minute Mcardle pacing test (to obtain maximal oxygen uptake VO_2max_) and fMRI scan. |
| ***Abbreviation:*** PAT, progressive aerobic training; FT, free training; IAT, Internet addiction test; QGU, questionnaire for gaming urges; IPAQ, international physical activity questionnaire. | | |

|  | |
| --- | --- |
| **S2.** The calculation formula for the step test. | |
| Item | Formula |
| Formula a | ${VO}_{2}=3.5+\left（ 0.2\times speed \right）+\left（ 0.9\times speed\times inclination\% \right）$ |
| Formula b | ${VO}_{2}=(Intensity of expectations)\%\times{VO}_{2max}$ |
| Male of formula | ${VO}_{2max}=111.33-\left（ 0.42\times HR \right）$ |
| Female of formula | ${VO}_{2max}=65.81-\left（ 0.1847\times HR \right）$ |
| ***Note:*** Inclination % refers to the percentage of incline on a treadmill and represents the vertical rise of an individual by x metres per 100 metres run, i.e. x%; Desired Intensity % is the percentage of oxygen uptake relative to maximal oxygen uptake (VO_2max_), and the ACSM guidelines recommend that the desired intensity of individuals who have not worked out regularly in the past should be set at a moderate intensity (46-63%) of aerobic training is optimal; HR is the heart rate (bpm).  **S3. Comparison of Baseline Characteristics Between Completers and Dropouts.**   \| Item(*M±SD*) \| Completers (*N*=64) \| Dropouts (*N*=8) \| *t* \| *p* \| \| --- \| --- \| --- \| --- \| --- \| \| Age \| 19.92±1.47 \| 20.38±1.52 \| -0.823 \| 0.413 \| \| IAT \| 62.91±12.93 \| 65.75±11.28 \| -0.596 \| 0.553 \| \| DSM-5 \| 5.22±2.07 \| 5.65±1.96 \| -0.546 \| 0.587 \| \| QGU \| 32.37±17.14 \| 35.20±15.89 \| -0.436 \| 0.664 \|   Abbreviation: IAT, Internet addiction test; DSM, Diagnostic and Statistical Manual of Mental Disorders-5; QGU, Questionnaire on gaming urge. | |
