# Supplementary figures and images for: Effects of Progressive Aerobic Training on Executive-Reward Network Connectivity and Symptoms of Internet Gaming Disorder: Randomized Controlled Trial
Source: JMIR Serious Games. 2025 Nov 28;13:e83597. doi: 10.2196/83597 (PMC12669919; doi:10.2196/83597)

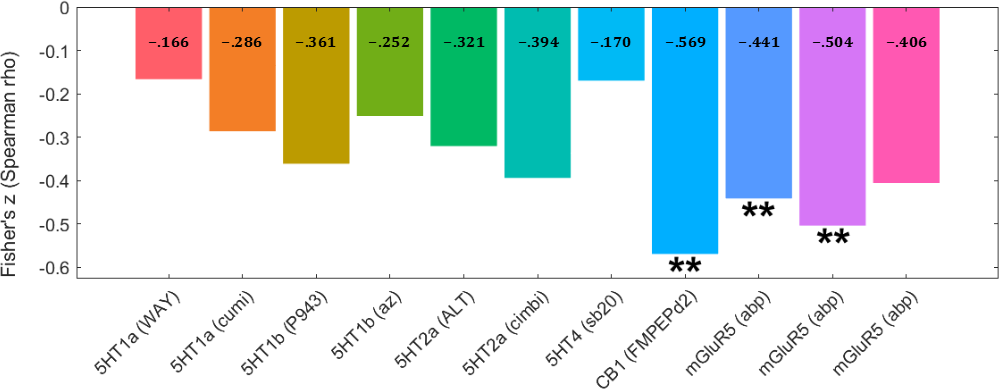

Supplement: Multimedia Appendix 2 [file games-v13-e83597-s002.png]
